# Ultrasound-Guided Quadratus Lumborum Block Versus Transversus Abdominis Plane Block for Laparoscopic Inguinal Hernia Repair and Appendicectomy Using Ropivacaine With Dexmedetomidine

**DOI:** 10.7759/cureus.33450

**Published:** 2023-01-06

**Authors:** Shashirajsinh S Vaghela, Manoj K Chaurasiya, Ravi Prakash, Mohammad Parvez Khan

**Affiliations:** 1 Department of Anesthesiology and Critical Care, King George’s Medical University, Lucknow, IND

**Keywords:** quadratus lumborum block, transversus abdominis plane block, ropivacaine, dexmedetomidine, hernia repair, laparoscopic, appendicectomy

## Abstract

Background

The present study aims to investigate the efficacy of ultrasound-guided quadratus lumborum (QL) block versus transversus abdominis plane (TAP) block for laparoscopic inguinal hernia repair and appendicectomy using ropivacaine with dexmedetomidine for quality and duration of postoperative analgesia.

Settings and design

This was a prospective, randomized, single-blind study conducted for one year (September 2020-August 2021) in the Department of Anesthesiology and Critical Care, King George’s Medical University, Lucknow, after obtaining ethical clearance from the institutional ethics committee.

Methods

A total of 64 patients of American Society of Anesthesiologists (ASA) grades I-II and ages between 20 and 50 years undergoing laparoscopic inguinal hernia repair and appendicectomy were randomly allocated into two groups of 32 each; group A received ultrasonography (USG)-guided quadratus lumborum block using 0.5% ropivacaine 20 ml with dexmedetomidine 0.5 mcg/kg of body weight, and group B was given USG-guided transversus abdominis plane block using 0.5% ropivacaine 20 ml with dexmedetomidine 0.5 mcg/kg of body weight after the induction of general anesthesia and before surgical incision. Data were analyzed using Student’s t-test, Mann-Whitney U test, and chi-square test as applicable.

Results

The duration of analgesia was statistically higher (P<0.001) in group A (21.00±3.73 hours) as compared to group B (14.44±2.99 hours). Group A had significantly less analgesic (P<0.001) at 12, 18, and 24 hours postoperatively. The visual analog scale (VAS) was significantly decreased in group A at rest and movement. The range of percentage changes in heart rate (HR) was significantly higher in group B as compared to group A at 12, 18, and 24 hours (group A: 7.23%-14.70%; group B: 6.41%-28.01%). The mean blood pressure (MBP) was significantly increased in group B at 12, 18, and 24 hours as compared to group A. The range of changes in baseline MBP in group A was less than in group B (group A: 0.73%-8.34%; group B: 0.73%-18.20%).

Conclusion

Quadratus lumborum block is effective and better than transversus abdominis plane block for providing postoperative analgesia during laparoscopic inguinal hernia repair and appendicectomy.

## Introduction

Alleviating pain during intraoperative and postoperative periods is one of the major concerns of anesthesiologists [1-3]. Infraumbilical surgeries such as laparoscopic inguinal hernia repair and appendicectomy have been associated with pain and hence morbidity. To relieve this pain, lots of modalities in the form of systemic analgesia, epidural analgesia, and various therapies have been employed, but presently, the trend is toward regional block. These blocks offer less systemic side effects with better analgesia.

Blind regional blocks have lower accuracy in placement and are associated with visceral injury and bleeding. The routine use of ultrasound improved the safety of the abdominal field block. Quadratus lumborum (QL) block and transversus abdominis plane (TAP) block have been found to be quite effective to relieve pain for infraumbilical surgeries such as laparoscopic inguinal hernia repair and appendicectomy. Transversus abdominis plane block via the triangle of Petit blocks the sensory nerves of the anterior abdominal wall before they leave this plane and pierces the musculature to innervate the entire anterior abdominal wall including the lower six thoracic and upper lumbar abdominal afferent (T7 to L1) [4]. Quadratus lumborum block provides superficial sensory, as well as deep somatic analgesia, by blocking lumbar nerve roots and nerves within TAP. Quadratus lumborum block relieves somatic pain, as well as visceral pain [5]. Quadratus lumborum block has been increasingly used for postoperative analgesia by extensive sensory suppression through a wide distribution of local anesthetics [6-8].

Dexmedetomidine is a highly selective α2 agonist, which provides sympatholytic, sedative, anxiolytic, hypnotic, and analgesic effects by decreasing sympathetic central nervous system outflow. Additionally, dexmedetomidine does not produce the side effects of opioids, such as nausea, vomiting, respiratory depression, urine retention, and pruritus [9]. Several local anesthetic agents have been used by anesthesiologists to provide effective and adequate postoperative analgesia. Ropivacaine belonging to the amino amide group has higher anesthetic potency with long-acting duration and less toxicity effect in comparison to bupivacaine. However, both drugs have similar pKa and plasma protein binding, but ropivacaine is less lipophilic than bupivacaine and has smaller volume of distribution, shorter elimination half-life, and greater clearance than bupivacaine in humans [10].

However, several randomized controlled trials have been conducted in recent years to compare the effects of transversus abdominis plane block to quadratus lumborum block in postoperative analgesia, but the results were inconsistent [11-15]. The aim of our randomized controlled trial was to compare quadratus lumborum block to transversus abdominis plane block for laparoscopic inguinal hernia repair and appendicectomy using ropivacaine with dexmedetomidine on the basis of following criteria: to assess the quality and duration of postoperative analgesia, hemodynamic effects, the need for rescue analgesics in the first 24 hours, side effects, and complications.

## Materials and methods

This was a prospective, randomized, single-blind study conducted for one year (September 2020-August 2021) after getting approval from the institutional ethics committee of the Department of Anesthesiology and Critical Care, King George’s Medical University, Lucknow (CTRI/2021/01/030498). Written informed consent was obtained from all the patients after explaining the details of the study in their language. Inclusion criteria were American Society of Anesthesiologists (ASA) grades I-II, ages between 20 and 50 years, patients for laparoscopic inguinal hernia repair and appendicectomy, and patients giving written consent. Exclusion criteria of the study were infection at the site of injection (inj), allergy to the study drug, localized malignant tumor, heart block, patients with coagulation disorder, difficulty in communication with patients, pregnant and lactating patients, obese patients, and technically difficult block.

Sample size

The sample size is calculated on the basis of variation in total analgesic consumption in two groups under study using the following formula: $n = \frac{\left ( z_\alpha + z_\beta \right )^{2} \left ( \sigma_{1}^{2} + \sigma_{2}^{2} \right )}{d^{2}}$, where $\sigma$_1_ = 1.55 and $\sigma$_2_ = 0.78, which are standard deviations of total analgesic consumption in two groups under the study of Kumar et al. [12]; d = mean ($\sigma$_1_, $\sigma$_2_), which is the minimum mean difference considered to be clinically significant; type I error α = 5%, corresponding to 95% confidence level; and type II error β = 10% for detecting results with 90% power of study. So, $n = \frac{\left ( 1.96 + 1.64 \right )^{2} \left ( 1.55^{2} + 0.78^{2} \right )}{1.165^{2}}$ = 29 (data loss factor = 10%). So, the minimum required sample size is *n *= 32 for each group.

Thus, a total of 64 patients participated in the present study based on the sample size calculation as explained above. Initially, we screened 70 patients taking into account the probable dropouts from the study. Six patients were excluded based on the exclusion criteria, and finally, 64 patients (32 patients in each group) were analyzed for data analysis. Randomization was done by the chit method. Sixty-four chits of both groups (group A and group B) were made by one of the investigators, and the investigator asked all the eligible 64 patients to choose any of the chit randomly before the induction of anesthesia, and patients were randomly allocated in any of the groups. All the patients were completely blinded about the interventions used during the study.

The current study was conducted on 64 patients of either sex, ages 20-50 years, and ASA grades I-II scheduled for elective laparoscopic inguinal hernia repair and appendicectomy under general anesthesia. Patients were divided into two groups by the chit method. Group A patients were administered quadratus lumborum (QL) block with 20 ml 0.5% ropivacaine with 0.5 mcg/kg body weight dexmedetomidine under ultrasound guidance. Group B patients were administered transversus abdominis plane (TAP) block with 20 ml 0.5% ropivacaine with 0.5 mcg/kg body weight dexmedetomidine under ultrasound guidance.

All patients were operated under general anesthesia. On the day of surgery, after confirming nil per orally (NPO) status of the patients, standard monitors were attached, and intravenous (IV) cannulation was done and IV fluid started. The patient was premedicated with inj ondansetron (0.1 mg/kg), inj midazolam (0.1 mg/kg), and inj fentanyl (2 mcg/kg). The patient was induced with inj propofol (2 mg/kg) and muscle relaxant inj vecuronium (0.1 mg/kg). The maintenance of anesthesia was done by nitrous oxide (N_2_O)/sevoflurane/oxygen (O_2_) (50% N_2_O, 49% O_2_, and 1% sevoflurane) and inj vecuronium (0.01 mg/kg). TAP/QL block was given after induction before incision.

Outcome measures of the study, e.g., hemodynamic parameters (heart rate {HR} and blood pressure), visual analog scale (VAS) score, and the need of rescue analgesia (inj paracetamol {pcm} {1 g IV}, first rescue analgesia; inj diclofenac {1 mg/kg IV}, second rescue analgesia) were recorded at postoperative six hours, 12 hours, 18 hours, and 24 hours. First inj paracetamol was administered when the VAS score was $\geq$3 or when the patient demanded it. However, inj paracetamol was kept at a frequency of every six hours (q6h). If the patient complained of significant pain within six hours of the administration of paracetamol, an injection of diclofenac was added. Complications such as nausea, vomiting, and bradycardia were monitored during the study.

Statistical analysis

All the collected data were entered in Microsoft Excel (Microsoft® Corp., Redmond, WA) and analyzed using Statistical Package for Social Sciences (SPSS) (IBM SPSS Statistics, Armonk, NY) version 23. Student’s t-test was used to analyze parametric data, while the Mann-Whitney U test was applied to nonparametric data, and chi-square test was applied to analyze categorical data. P values of <0.05 were considered statistically significant.

## Results

**Figure 1:**
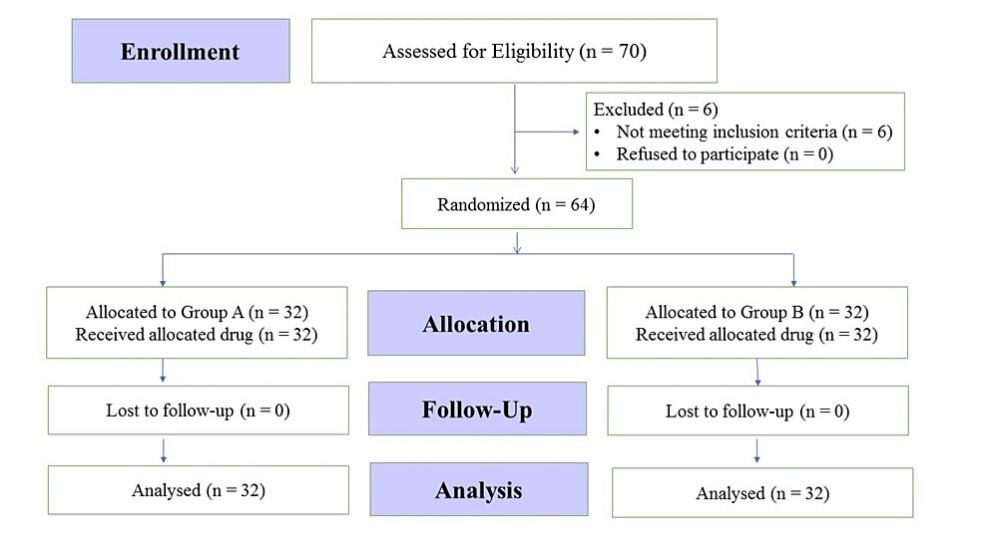
CONSORT flow diagram n, number of patients; CONSORT: Consolidated Standards of Reporting Trials

Sixty-four patients were randomly assigned in two groups (32 patients in each group) in the present study. Figure 1 shows the Consolidated Standards of Reporting Trials (CONSORT) diagram depicting the participation and recruitment of patients in the study.

There were no significant differences (P>0.05) observed between groups A and B in respect to demographic data (age and sex) and physical data (weight and ASA grade) as displayed in Table 1 and Table 2, respectively.

**Table 1:** Comparison of demographic profile between two groups N, number of patients; %, percentage of patients

Serial number	Demographic characteristics	Group A (N=32)		Group B (N=32)		Total (N=64)	
1	Mean age±SD (range) in years	39.50±10.22 (21-50)		34.81±9.49 (18-50)		37.16±10.12 (18-70)	
	Student’s t-test	t=1.891; P=0.063					
2	Gender	N	%	N	%	N	%
	Female	6	18.8	5	15.6	11	17.2
	Male	26	81.3	27	84.4	53	82.8
	Chi-square test	Chi-square=0.110; P=0.740					

**Table 2:** Comparison of physical profile between two groups N, number of patients; %, percentage of patients; ASA, American Society of Anesthesiologists

Serial number	Physical characteristics	Group A (N=32)		Group B (N=32)		Total (N=64)	
1	Mean weight±SD (range) in kg	58.69±5.70 (50-70)		58.00±5.17 (50-70)		58.39±5.41 (50-70)	
	Student’s t-test	t=0.574; P=0.568					
2	ASA grade	N	%	N	%	N	%
	ASA grade I	7	21.9	11	34.4	18	28.1
	ASA grade II	25	78.1	21	65.6	46	71.9
	Chi-square test	Chi-square=1.237; P=0.266					

The heart rate of patients in both groups A and B was found to be comparable at baseline and thereafter at all the periods of observation until six hours after block. The heart rate of patients was increased significantly (P<0.001) in group B as compared to group A at 12 hours, 18 hours, and 24 hours (Table 3).

**Table 3:** Comparison of heart rate between two groups at different time intervals N, number of patients; ind, induction; Imm, immobilization

Serial number	Time period	Group A (N=32)		Group B (N=32)		Student’s t-test	
		Mean	SD	Mean	SD	t	P
1	Before ind (baseline)	77.81	10.23	77.53	8.87	0.118	0.907
2	After ind	84.13	7.43	83.81	6.99	0.173	0.863
3	Before block	83.44	5.00	82.50	3.70	0.852	0.397
4	After block	85.06	4.74	85.25	4.87	-0.156	0.876
5	Imm (zero hours)	85.63	5.68	85.50	4.52	0.097	0.923
6	6 hours	84.69	5.93	86.38	4.47	-1.285	0.204
7	12 hours	86.06	5.89	94.31	8.40	-4.550	<0.001
8	18 hours	87.94	6.99	99.25	6.15	-6.870	<0.001
9	24 hours	89.25	9.56	98.94	6.24	-4.799	<0.001

The mean blood pressure (MBP) of both groups was comparable at baseline and thereafter at all the periods of observation until six hours. The mean blood pressure of group B patients was found significantly higher (P<0.001) than group A at 12 hours, 18 hours, and 24 hours (Table 4).

**Table 4:** Comparison of mean blood pressure between two groups at different time intervals N, number of patients; ind, induction; Imm, immobilization

Serial number	Time period	Group A (N=32)		Group B (N=32)		Student’s t-test	
		Mean	SD	Mean	SD	t	P
1	Before ind (baseline)	72.72	7.04	70.38	5.50	1.484	0.143
2	After ind	72.81	5.58	70.91	5.84	1.335	0.187
3	Before block	73.25	3.52	73.59	4.88	-0.323	0.748
4	After block	75.03	4.58	75.19	2.55	-0.169	0.866
5	Imm (zero hours)	76.91	5.18	77.81	2.42	-0.897	0.373
6	6 hours	74.50	2.74	75.84	3.28	-1.779	0.080
7	12 hours	75.16	3.20	79.38	3.51	-5.023	<0.001
8	18 hours	78.78	3.85	82.38	2.50	-4.430	<0.001
9	24 hours	77.69	4.78	83.63	3.33	-5.770	<0.001

There was a significant increase (P<0.001) in mean VAS in group B patients as compared to group A at six hours, 12 hours, 18 hours, and 24 hours. No pain was observed at zero hours (immobilization postoperative) in any of the patients. The pain of both groups A and B was comparable at six hours, 12 hours, 18 hours, and 24 hours (Table 5).

**Table 5:** Comparison of pain (VAS score) between two groups at different time intervals VAS, visual analog scale; N, number of patients

Serial number	Time	Group A (N=32)			Group B (N=32)			Mann-Whitney U test	
		Median	Mean	SD	Median	Mean	SD	Z	P
1	0 hours	0.00	0.00	0.00	0.00	0.00	0.00	0.000	1.000
2	6 hours	0.00	0.13	0.49	0.00	0.31	0.86	-0.903	0.366
3	12 hours	0.00	0.38	1.07	4.00	3.25	1.88	-5.542	<0.001
4	18 hours	1.00	1.47	1.63	4.00	3.72	1.14	-4.896	<0.001
5	24 hours	2.00	2.22	1.70	3.50	3.88	1.45	-3.810	0.001

Patients of group B required the first dose of rescue analgesia significantly earlier than that of group A (14.44±2.99 versus 21.00±3.73 hours) (P<0.001) (Table 6).

**Table 6:** Comparison of rescue dose requirement between two groups N, number of patients; %, percentage of patients; pcm, paracetamol; IV, intravenous

Serial number		Group A (N=32)		Group B (N=32)		Total (N=64)	
1	Time of first dose of rescue analgesia required±SD (range) hours	21.00±3.73 (12-24)		14.44±2.99 (12-18)		17.72±4.71 (12-24)	
	Student’s t-test	t=7.758; P<0.001					
2	Total doses of rescue analgesia	N	%	N	%	N	%
	Nil	14	43.8	0	0.0	14	21.9
	1 g pcm IV	15	46.9	17	53.1	32	50.0
	2 g pcm IV	0	0.0	3	9.4	3	4.7
	1 g pcm+75 mg diclofenac	3	9.4	12	37.5	15	23.4
	Chi-square test	Chi-square=22.525; P<0.001					

Group A patients need significantly less rescue analgesia than group B patients. The difference in number/type of rescue analgesia required by two groups was found to be significant statistically (P<0.001). No complications were noted after giving QL block and TAP block such as nausea, vomiting, dizziness, and infection.

## Discussion

It is the responsible task of all anesthesiologists to manage pain during intraoperative and postoperative periods of surgeries. Several forms of analgesia have been recommended to attenuate intraoperative and postoperative periods of pain during surgeries. We recommend that quadratus lumborum block is better than transversus abdominis plane block for providing postoperative analgesia during laparoscopic inguinal hernia repair and appendicectomy. In the present study, the duration of analgesia was maximum in the group receiving QL block (21.00±3.73 hours) followed by the group receiving TAP block (14.44±2.99 hours), and the intergroup difference was found to be significant. On exploring the intergroup differences, it was observed that the group receiving QL block had significantly longer duration of analgesia as compared to the group receiving TAP block.

The findings of our current study are consistent with the findings of several previous studies. Our findings are in accordance with a study by Verma et al. who concluded that quadratus lumborum block provides prolonged and effective analgesia in comparison to TAP block up to 72 hours post-cesarean section [16]. In another study on patients undergoing total abdominal hysterectomy, Yousef observed similar results. Patients in the group receiving QL block consumed significantly less fentanyl and morphine than patients in the TAP group, VAS for pain was significantly higher in the TAP group than in the group receiving QL block at all times (3.2±0.43 for TAP block and 1.9±0.32 for QL block), the duration of postoperative analgesia was shorter in the TAP group than in the group receiving QL block, and the number of patients who requested analgesia was significantly higher in the TAP group than in the group receiving QL block [15].

In the present study, no rescue analgesia was required by 43.8% of patients of the group receiving QL block, 46.9% required only 1 g paracetamol intravenous (pcm IV), and 9.4% required 1 g pcm with 75 mg diclofenac IV, while all the patients of the group receiving TAP block required rescue analgesia, 53.1% required only 1 g pcm IV, 9.5% required 2 g pcm IV, and 37.5% required 1 g pcm with 75 mg diclofenac IV. Data represented in the current study showed that there is a significant difference in intraoperative and postoperative heart rate (HR) and mean blood pressure (MBP). In the group receiving QL block, the range of percentage change in baseline heart rate was less than the group receiving TAP block. The heart rate of patients of the group receiving TAP block was significantly higher than the group receiving QL block (for the group receiving QL block: 7.23%-14.70%; for the group receiving TAP block: 6.41%-28.01%). We found a significant reduction in the mean blood pressure of the group receiving QL block in comparison to the group receiving TAP block. The range of change in baseline MBP in the group receiving QL block was less than the group receiving TAP block (for the group receiving QL block: 0.73%-8.34%; for the group receiving TAP block: 0.73%-18.20%).

Deng et al., in their study conducted on patients undergoing laparoscopic colorectal surgery, concluded that the QL block is a more effective postoperative analgesia because it reduces sufentanil consumption as compared to TAP block [17]. Malla et al. reviewed studies involving 188 patients comparing QL block to TAP block for postoperative pain management in abdominal surgery. They concluded that QL blockade leads to a significant reduction in 24-hour morphine consumption and postoperative pain scores, with no increase in adverse event rates, and concluded that QL blockade is likely a preferable regional analgesic technique than TAP blockade [18]. Similar result was reported by Liu et al. who reviewed patients undergoing abdominal surgery while comparing quadratus lumborum block to transversus abdominis plane block for postoperative analgesia [19]. In a study conducted by Li et al. in 2019, they concluded that preoperative lateral TAP block did not decrease intra- and postoperative opioid consumption, nor did it relieve pain intensity or promote postoperative recovery in the first 24 hours after surgery in patients undergoing retroperitoneoscopic renal surgery [20].

The findings of the present study are also consistent with the findings of the meta-analysis published in 2020, which concluded that quadratus lumborum block resulted in better intraoperative and postoperative analgesia with a smaller number of patients requiring analgesia postoperatively, less morphine consumption, less fentanyl consumption, reduced VAS score at 24 hours postoperatively, and decreased incidence of dizziness in comparison to TAP block. In addition, quadratus lumborum block also reduces the duration of anesthesia, operative time, and the incidence of nausea and vomiting in comparison to TAP block [21].

In our study, we did not notice any complications such as nausea, vomiting, dizziness, and infection after giving QL block and TAP block. Our present study found that the duration of analgesia was better in the group receiving QL block as compared to the group receiving TAP block. Rescue analgesic requirements were less in the group receiving QL block in comparison to the group receiving TAP block. Hemodynamics was better controlled in the group receiving QL block and the group receiving TAP block.

This study was limited to infraumbilical laparoscopic surgery only, so its result may not be applicable to other types of surgeries. Due to the small sample size, the detection of differences in uncommon side effects may not be accurate. It was limited to patients whose operation was carried out under general anesthesia only, so the finding may or may not be extrapolated to surgery in regional anesthesia. Patients were not given any basal analgesics other than block until rescue analgesics. Further, the types of surgeries were not the same.

## Conclusions

The present study concluded that quadratus lumborum block has several beneficial effects as compared to transversus abdominis plane block using ropivacaine with dexmedetomidine in laparoscopic inguinal hernia repair and appendicectomy in terms of better postoperative analgesia, better pain score (VAS score), longer duration of postoperative analgesia, requirement of first rescue analgesia after a longer time, less total rescue analgesia consumption, and better hemodynamic stability (heart rate and mean blood pressure).
